# A Photomodulable Bacteriophage‐Spike Nanozyme Enables Dually Enhanced Biofilm Penetration and Bacterial Capture for Photothermal‐Boosted Catalytic Therapy of MRSA Infections

**DOI:** 10.1002/advs.202301694

**Published:** 2023-06-13

**Authors:** Haibin Wu, Min Wei, Shen Hu, Pu Cheng, Shuhan Shi, Fan Xia, Lenan Xu, Lina Yin, Guang Liang, Fangyuan Li, Daishun Ling

**Affiliations:** ^1^ School of Pharmaceutical Sciences Hangzhou Medical College Hangzhou 311399 P. R. China; ^2^ Institute of Pharmaceutics College of Pharmaceutical Sciences Zhejiang University Hangzhou 310058 P. R. China; ^3^ Department of Obstetrics and Gynaecology The Second Affiliated Hospital School of Medicine Zhejiang University Hangzhou 310009 P. R. China; ^4^ Institute of Innovative Medicine College of Pharmaceutical Sciences Zhejiang University Hangzhou 310012 P. R. China; ^5^ World Laureates Association (WLA) Laboratories Shanghai 201203 P. R. China; ^6^ Frontiers Science Center for Transformative Molecules School of Chemistry and Chemical Engineering National Center for Translational Medicine Shanghai Jiao Tong University Shanghai 200240 P. R. China

**Keywords:** antibacterial therapy, bacterial capture, biofilm penetration, nanozyme, nature‐inspired nanostructures

## Abstract

Nanozymes, featuring intrinsic biocatalytic effects and broad‐spectrum antimicrobial properties, are emerging as a novel antibiotic class. However, prevailing bactericidal nanozymes face a challenging dilemma between biofilm penetration and bacterial capture capacity, significantly impeding their antibacterial efficacy. Here, this work introduces a photomodulable bactericidal nanozyme (ICG@hMnO*_x_*), composed of a hollow virus‐spiky MnO*_x_* nanozyme integrated with indocyanine green, for dually enhanced biofilm penetration and bacterial capture for photothermal‐boosted catalytic therapy of bacterial infections. ICG@hMnO*_x_* demonstrates an exceptional capability to deeply penetrate biofilms, owing to its pronounced photothermal effect that disrupts the compact structure of biofilms. Simultaneously, the virus‐spiky surface significantly enhances the bacterial capture capacity of ICG@hMnO*_x_*. This surface acts as a membrane‐anchored generator of reactive oxygen species and a glutathione scavenger, facilitating localized photothermal‐boosted catalytic bacterial disinfection. Effective treatment of methicillin‐resistant *Staphylococcus aureus*‐associated biofilm infections is achieved using ICG@hMnO*_x_*, offering an appealing strategy to overcome the longstanding trade‐off between biofilm penetration and bacterial capture capacity in antibacterial nanozymes. This work presents a significant advancement in the development of nanozyme‐based therapies for combating biofilm‐related bacterial infections.

## Graphical abstract

A photomodulable bacteriophage‐spike bactericidal nanozyme (ICG@hMnO*_x_*) composed of hollow virus‐spiky MnO*_x_* nanozyme and indocyanine green is constructed to exhibit dually enhanced biofilm penetration and bacterial capture for catalytic therapy of bacterial infections. This unique construct addresses the long‐standing trade‐off between biofilm penetration and bacterial capture capacity of antibacterial nanozymes and offers a promising strategy for catalytic therapy of bacterial infections.

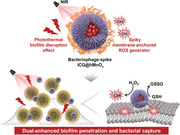

## 1 Introduction

The discovery of antibiotics has enabled the successful treatment of previously incurable bacterial infections, saved countless lives and improved the quality of life.^[^ ^1^ ^]^ However, the emergence and spread of antimicrobial resistance has become a global crisis of public health, which greatly increases the risk of morbidity and death.^[^ ^2^ ^]^ Among them, methicillin‐resistant Staphylococcus aureus (MRSA) is recognized as a priority pathogen that poses a serious threat to human health by the World Health Organization (WHO).^[^ ^2b^ ^]^ Due to its antibiotic resistance and ability to form biofilms, MRSA has become a leading cause of both community‐ and hospital‐acquired infections, commonly associated with soft tissue infections, osteomyelitis, and hospital‐acquired infections.^[^ ^3^ ^]^ Apart from the acquired resistance, bacterial cells can also produce extracellular polymeric substances (EPS) to form biofilm.^[^ ^4^ ^]^ The clinical challenge is significantly amplified once bacteria form biofilms, which can act as a barrier against most currently available antibiotics.^[^ ^5^ ^]^ Furthermore, the biofilm can alter the phenotype of encapsulated bacteria cells and induce the formation of persistent bacteria cells.^[^ ^4a,b^ ^]^ Multiple or high doses of antibiotic agents are usually required for the eradication of resistant bacteria cells. However, overuse of antibiotics can induce severe side effects,^[^ ^6^ ^]^ such as impairment of immune system, kidney toxicity, and digestive system disorders. Worse still, novel resistance mechanisms can be developed by the long‐term use and/or overuse of antibiotics.^[^ ^7^ ^]^ Given how fast the bacterial resistance has evolved and the difficulty in developing novel antibiotics, innovative “outside of the box” strategies are urgently needed to combat resistant bacteria infections.^[^ ^8^ ^]^

Recent development of nanocatalytic therapy (NCT) shows great promise for the treatment of refractory bacterial infections.^[^ ^9^ ^]^ By producing highly toxic reactive oxygen species (ROS) such as hydroxyl radicals (·OH), NCT can potentially overcome bacterial resistance to conventional antibiotics.^[^ ^9^, ^10^ ^]^ However, the toxic ROS are known to have very short lifetimes, thus leading to a limited radius of action that can only cause oxidative damage to surrounding substances.^[^ ^11^ ^]^ Therefore, surface engineering of nanozymes with enhanced bacterial adhesion is highly desirable.

Nature has long been a source of inspiration for material scientists.^[^ ^12^ ^]^ Over millions of years of evolution, the majority of bacteriophage viruses have evolved to possess multiple tail spikes, allowing the effective capture of pathogenic bacteria.^[^ ^13^ ^]^ Inspired by the fascinating structure of bacteriophage viruses, spiky nanozymes with a strong adhesive capacity to bacteria have been developed for combating bacterial infection.^[^ ^14^ ^]^ Qiu and co‐workers reported a hybrid spiky nanozyme composed of a metal‐organic framework (MOF)‐derived ROS catalytic core and mesoporous‐silica‐based spiky shell for antibacterial therapy.^[^ ^14a^ ^]^ Similarly, Qu et al. designed a hybrid MOF@COF bactericidal nanozyme, whereby the NH_2_‐MIL‐88B iron MOF‐based nanozyme was encapsulated in the spiky covalent organic frameworks (COF) shell.^[^ ^14b^ ^]^ These virus‐like spiky silica or COF shells are coated onto the surface of nanozymes for efficient bacterial capture.^[^ ^14^ ^]^ Ideally, nanozymes with an intrinsic spiky structure that enables more direct interaction with bacteria are highly beneficial for enhanced bactericidal performance.^[^ ^15^ ^]^ However, these spiky nanozymes have an inherent trade‐off between their biofilm penetration and bacterial capture capacities. According to the Brownian Dynamics theory, the motion of particles is inversely proportional to the friction force.^[^ ^16^ ^]^ Therefore, the intrinsic spiky structure greatly restricts the penetration of nanozymes in the biofilm, which is mainly achieved by nanozymes with a small size and smooth surface.^[^ ^17^ ^]^ Due to the poor biofilm penetration capacity, such spiky antibacterial nanozymes are challenging to use in the treatment of deep biofilm infections. Although biofilm penetration and bacterial capture can be respectively achieved by nanozymes, the contradiction between particle motion and adhesion makes it difficult for nanozymes to exhibit both activities simultaneously.

**Scheme 1:**
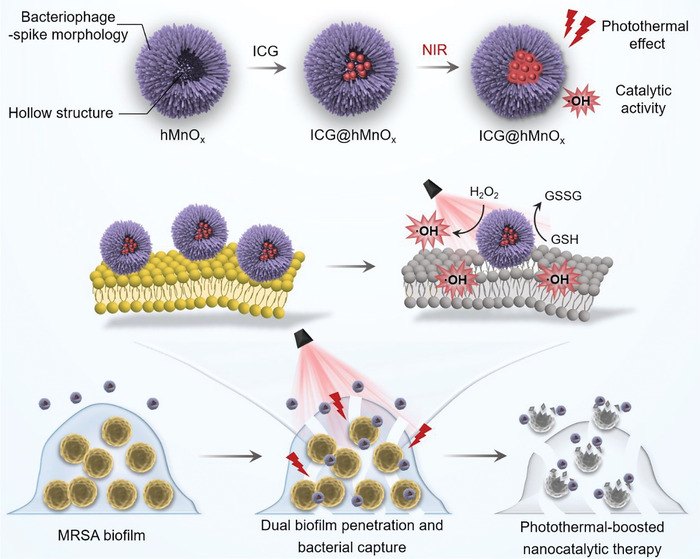
Schematic illustration of the photomodulable bacteriophage‐spike nanozyme for photothermal‐boosted catalytic therapy against MRSA biofilm infection. The ICG integrated hollow virus‐spiky MnO*_x_* nanozyme exhibits both photothermal and catalytic activities. The biofilm disruption facilitated by the photothermal effect allows for deep penetration of ICG@hMnO*_x_*, which can then act as a bacteriophage‐mimetic membrane‐anchored ROS generator. The ICG@hMnO*_x_* enables dually enhanced biofilm penetration and bacterial capture to treat MRSA biofilm‐associated infections.

To overcome the dilemma, we herein report the designed fabrication of a bacteriophage‐spike nanozyme ICG@hMnO*_x_* with dually enhanced biofilm penetration and bacterial capture capacities. The hollow hMnO*_x_* nanozyme with an intrinsic bacteriophage‐spike surface is synthesized via a facile template etching process using stöber silica nanoparticle (SSN) as a self‐sacrificing template and potassium permanganate (KMnO_4_) as a metal precursor. By equipping the hMnO*_x_* nanozyme with photothermal agent indocyanine green (ICG), ICG@hMnO*_x_* with photothermal effect and a virus‐spiky topological structure has been successfully fabricated, enabling a photothermal‐boosted localized “capture and killing” action against biofilm‐embedded MRSA. Given the short life and limited diffusion radius of ROS,^[^ ^11^ ^]^ the MRSA‐capturing ICG@hMnO*_x_* provides massive possibilities for oxidative destruction of membrane and intracellular components of MRSA. Meanwhile, highly efficient depletion of glutathione (GSH) can also be achieved by ICG@hMnO*_x_*, thus further weakening the bacterial defense system and enhancing the sterilization effect of ROS. More importantly, in addition to the photothermal‐boosted enzyme‐mimetic catalytic activities, the spatiotemporal programmable heating can also disrupt the extracellular matrix of the MRSA biofilm,^[^ ^18^ ^]^ thereby greatly promoting the deep biofilm penetration of ICG@hMnO*_x_* (**Scheme** **1**). To the best of our knowledge, this is the first antibacterial nanozyme whose biofilm penetration and bacterial capture capacities can be dually reinforced, making it a critical step to overcome this longstanding dilemma between biofilm penetration and bacterial capture.

## 2 Results and Discussion

### 2.1 Fabrication and Structural Characterization of ICG@hMnO*_x_*

**Figure 1:**
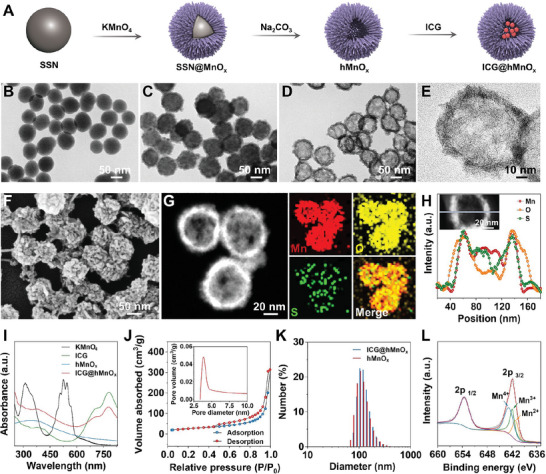
Fabrication and characterization of the photomodulable bacteriophage‐spike ICG@hMnO*_x_*. A) Schematic illustration of the synthetic procedure of ICG@hMnO*_x_*. TEM images of the B) SSN, C) SSN@MnO*_x_*, and D) ICG@hMnO*_x_*. E) The HRTEM image of a single ICG@hMnO*_x_*. F) The SEM image of the ICG@hMnO*_x_*. G) Dark‐field STEM and the corresponding EDX mapping (Mn, O, S) images of the ICG@hMnO*_x_*. H) Elemental line scanning profile of the ICG@hMnO*_x_*. I) UV–vis absorption spectra of indicated samples. J) Nitrogen absorption‐desorption isotherm with the corresponding pore‐size distribution of the ICG@hMnO*_x_*. K) DLS characterization of ICG@hMnO*_x_* and hMnO*_x_*. L) High‐resolution XPS spectra of *Mn 2p* in ICG@hMnO*_x_*.

In the present study, we report a photomodulable bacteriophage‐spike nanozyme ICG@hMnO*_x_* that enables dual biofilm penetration and bacterial capture for photothermal‐boosted catalytic therapy of MRSA biofilm infection. The procedure for the synthesis of ICG@hMnO*_x_* is presented in **Figure** **1**A. The hollow virus‐spiky nanozyme hMnO*_x_* was prepared by a sacrificial templating method.^[^ ^19^ ^]^ First, SSN with a smooth surface was synthesized via a stöber method (Figure 1B). Then, a virus‐spiky MnO*_x_* shell was grown on the surface of SSNs (Figure 1C), and the SSN core was etched by Na_2_CO_3_ solution to obtain the hMnO*_x_*. An FDA‐approved photothermal agent ICG was integrated into the hMnO*_x_* to fabricate ICG@hMnO*_x_*. The transmission electron microscopy (TEM) image and high‐resolution transmission electron microscopy (HRTEM) image show that the ICG@hMnO*_x_* exhibits a bacteriophage‐like hollow morphology with porous and rough surface (Figure 1D,E). As demonstrated in Figure 1F and Figure S1, Supporting Information, the scanning electron microscopy (SEM) images clearly show randomly oriented spikes with a high density on the surface of ICG@hMnO*_x_*. The scanning transmission electron microscopy (STEM) and energy dispersive X‐ray spectroscopy (EDX) elemental scanning reveal the distribution of Mn, O, and S atoms in ICG@hMnO*_x_*, demonstrating the integration of sulfur‐containing ICG (Figure 1G,H), which is also verified by the characteristic absorbance peaks of ICG in Ultraviolet and visible (UV–vis) spectroscopy of ICG@hMnO*_x_* (Figure 1I). As shown in Figure S2, Supporting Information, the loading capacity of ICG is calculated to be 63.05% according to the Lambert‐beer's law. The efficient integration of ICG is attributed to the highly mesoporous nature of ICG@hMnO*_x_* as evidenced by the N_2_ adsorption/desorption isotherms and corresponding pore‐size distribution (Figure 1J). Dynamic light scattering (DLS) result and *ζ*‐potential measurement show that ICG@hMnO*_x_* is negatively charged and well‐dispersed in water with a hydrodynamic diameter of ≈106 nm and a zeta potential of −33 mV (Figure 1K and Figure S3, Supporting Information). Additionally, it is worth noting that ICG@hMnO*_x_* displays the characteristic Tyndall effect and maintains its hydrodynamic diameter in various environments, including deionized water, phosphate‐buffered saline (PBS), and Dulbecco's modified Eagle medium (DMEM), which indicates excellent colloidal stability (Figure S4, Supporting Information). Besides, the ICG@hMnO*_x_* show negligible ICG leakage within 3 days (Figure S5, Supporting Information), indicating the stable loading of ICG in the nanozyme. The X‐ray photoelectron spectroscopy (XPS) spectrum of ICG@hMnO*_x_* shows the co‐existence of Mn^2+^, Mn^3+^, and Mn^4+^ (binding energy at 641.1, 641.9, and 642.7 eV, respectively), and the X‐ray diffraction as well as the selected area electron diffraction ring pattern demonstrate the multiple crystal phases of hMnO*_x_* (Figure 1L; Figures S6 and S7, Supporting Information) for potential redox reaction with endogenous GSH and Fenton‐like reactions.^[^ ^20^ ^]^

### 2.2 Laser‐Augmented Catalytic Performance of ICG@hMnO*_x_*. Antibacterial Therapy; Bacterial Capture; Biofilm Penetration; Nanozyme; Nature‐Inspired Nanostructures

**Figure 2:**
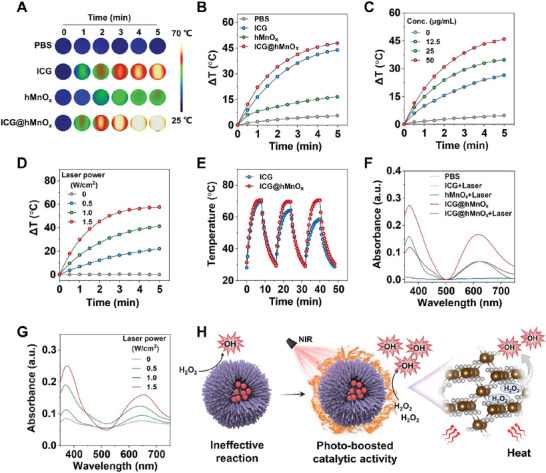
Laser‐enhanced POD‐like activity of the photomodulable bacteriophage‐spike ICG@hMnO*_x_*. A) Time‐dependent infrared thermal images and B) heating curves of aqueous dispersions of indicated samples under the irradiation with an 808 nm laser (1.0 W cm^−2^). C) Temperature elevation curves of ICG@hMnO*_x_* at different concentrations under irradiation with an 808 nm laser (1.0 W cm^−2^). D) Heating curves of ICG@hMnO*_x_* under 808 nm laser irradiation with different power densities. E) Temperature evolutions of ICG and ICG@hMnO*_x_* solutions subjected to laser on‐off cycles under 808 nm laser irradiation (1.0 W cm^−2^). F) UV–vis absorption spectra of 3,3′,5,5′‐tetramethylbenzidine (TMB) solution containing different samples with or without NIR‐808 nm laser irradiation (1.0 W cm^−2^). G) UV–vis absorption spectra of TMB solution with ICG@hMnO*_x_* upon 808 nm laser irradiation at different power densities (0–1.5 W cm^−2^). H) Schematic illustration of the laser‐augmented POD‐like activities of the ICG@hMnO*_x_* nanozyme.

First, the temperature evolution upon near‐infrared (NIR) irradiation was recorded to evaluate the photothermal performance of ICG@hMnO*_x_*. As shown in the infrared thermal images and photothermal curves (**Figure** **2**A,B), compared with hMnO*_x_*, ICG@hMnO*_x_* exhibits a significant time‐dependent temperature increase when exposed to a NIR laser, suggesting that the integration of ICG successfully enables the photothermal activity of ICG@hMnO*_x_*. The concentration‐ and power density‐dependent photothermal effect when exposed to an 808 nm laser further confirms the NIR photothermal transduction efficiency of ICG@hMnO*_x_* (Figure 2C,D). Besides, unlike the rapid decay in the photothermal activity of free ICG, the plateau temperature of ICG@hMnO*_x_* exhibits a negligible change during the laser on/off cycles (Figure 2E), suggesting the excellent photostability of ICG@hMnO*_x_* due to the declined oxidation and degradation of the ICG trapped inside the pores of ICG@hMnO*_x_*.^[^ ^21^ ^]^

According to the Arrhenius equation, the rate of a chemical reaction is positively associated with the reaction temperature. The heat energy can promote catalytic reaction by overcoming the activation barrier and improving the mass transfer rate.^[^ ^22^ ^]^ Encouraged by the excellent photothermal transduction efficiency, the peroxidase (POD)‐like activity of ICG@hMnO*_x_* was further investigated. Under the irradiation of an 808 nm laser, the POD‐like activity of ICG@hMnO*_x_* is significantly enhanced, whereas hMnO*_x_* shows a negligible catalytic activity enhancement (Figure 2F). These results suggest that the ICG mediates the photothermal‐boosted catalytic activity of ICG@hMnO*_x_*. Moreover, the enhancement of the POD‐like activity of the ICG@hMnO*_x_* displays a laser power density‐dependent pattern, indicating the catalytic activity of ICG@hMnO*_x_* can be remotely tuned (Figure 2G). As shown in the electron paramagnetic resonance (EPR) spectra, the DMPO‐OH signals demonstrate the generation of free radical species •OH by ICG@hMnO*_x_* under laser irradiation, which further confirms the photothermal‐boosted POD‐like activity of ICG@hMnO*_x_* (Figure S8, Supporting Information). The direct effect of thermal energy on the POD‐like activity of ICG@hMnO*_x_* was also investigated. As shown in the Figure S9, Supporting Information, the thermal effect can efficiently promote the catalytic reaction of ICG@hMnO*_x_*. Overall, these results suggest that the catalytic activity of ICG@hMnO*_x_* can be regulated by the NIR laser irradiation (Figure 2H).

### 2.3 Anti‐Bacterial and Anti‐Biofilm Performance of ICG@hMnO*_x_*

**Figure 3:**
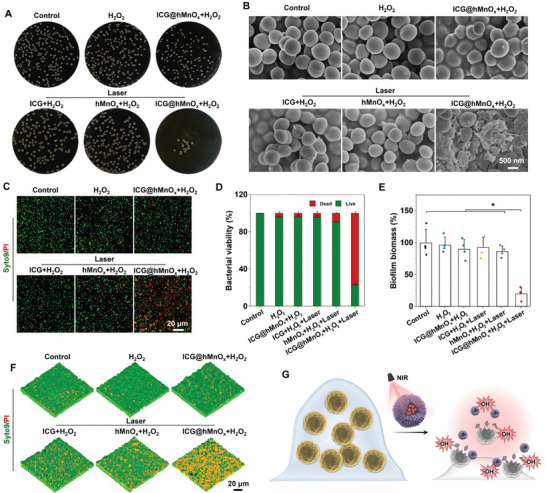
Anti‐bacterial and anti‐biofilm performance of the photomodulable bacteriophage‐spike ICG@hMnO*_x_*. A) Representative plates of MRSA colonies after different treatments. B) SEM images of MRSA under different treatments. C) Confocal laser scanning microscope (CLSM) images of live/dead staining, in which green fluorescence indicates the live bacteria while red fluorescence indicates dead bacteria. D) MRSA viability determined by the live/dead ratio (*n* = 4). E) Biomass of MRSA biofilm after indicated treatments (*n* = 4). F) 3D CLSM images of live/dead stained MRSA biofilm after different treatments. G) Schematic illustration of the highly efficient bactericidal effect by ICG@hMnO*_x_*. Data are presented as mean ± s.d. *p < 0.05.

Encouraged by the photothermal‐boosted nanocatalytic activities of ICG@hMnO*_x_*, their antibacterial effects were further evaluated. The antibiotic‐resistant MRSA was chosen as a model strain to investigate the bactericidal activity of ICG@hMnO*_x_*.^[^ ^2b^ ^]^ The antibacterial activity of ICG@hMnO*_x_* was first investigated via a colony counting method. In contrast with control groups that merely exhibit moderate antibacterial activities, ICG@hMnO*_x_* coupled with laser treatment can significantly reduce the colony forming units (CFU) benefitting from the photothermal‐boosted catalytic effect (**Figure** **3**A). Moreover, the cytosolic membranes of MRSA cells are extensively damaged in the ICG@hMnO*_x_* plus laser irradiation group, while minimal morphological changes are observed in other groups (Figure 3B). As expected, the synergistic combination of nanocatalytic effect and laser irradiation mediated by the ICG@hMnO*_x_* leads to the most severe bacterial elimination against MRSA with a minimum inhibitory concentration (MIC) of 50 µg mL^−1^ and a minimum bactericidal concentration (MBC) of 50 µg mL^−1^, respectively (Figure 3C,D and Figure S10, Supporting Information). It is worth noting that the photothermal effect alone has minimal antibacterial activity, and the antibacterial effect of ICG@hMnO*_x_* is largely attributed to its POD‐like catalytic activity for efficient ROS generation (Figure S11, Supporting Information). The photomodulable bactericidal effect provides a promising strategy for precise therapy of infectious diseases. Moreover, the bactericidal activity of ICG@hMnO*_x_* can be precisely regulated by changing the NIR laser irradiation time (Figure S12, Supporting Information). Therefore, the ICG@hMnO*_x_* exhibits a unique combination of features, including biofilm penetration and bacterial capture, precisely tunable antibacterial activity, and GSH depletion, which make it unique among the state‐of‐the‐art antibacterial nanomaterials such as silver nanoparticles.

Compared to planktonic bacteria, pathogens located within the biofilm matrix are inherently tolerant to most antibiotics.^[^ ^4^, ^23^ ^]^ Notably, the crystal violet staining reveals that ICG@hMnO*_x_* plus laser irradiation treatment significantly reduces the MRSA biofilm biomass as compared with other treatments (Figure 3E). Consistently, the ICG@hMnO*_x_* plus laser irradiation treatment mediates the most serious damage to MRSA biofilm as indicated by the most bacteria dead inside biofilm in the live/dead fluorescence staining assay (Figure 3F). The above results collectively demonstrate that the photothermal‐boosted NCT enabled by ICG@hMnO*_x_* not only combats the planktonic bacteria but also efficiently eradicates the MRSA biofilms (Figure 3G). Moreover, ICG@hMnO*_x_* exhibits negligible hemolytic activity and cytotoxicity, suggesting the good cellular biocompatibility of ICG@hMnO*_x_* (Figure S13, Supporting Information).

**Figure 4:**
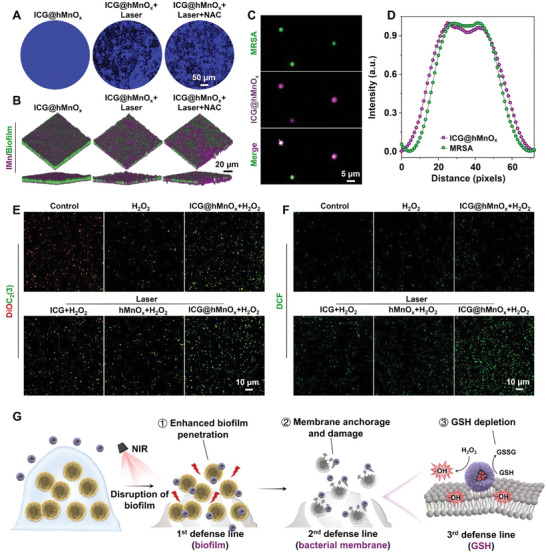
Concurrent biofilm penetration and bacterial capture capacity of the photomodulable bacteriophage‐spike ICG@hMnO*_x_*. A) Crystal violet‐stained biofilm with or without 808 nm laser (1.0 W cm^−2^) irradiation. B) 3D CLSM images of the ICG@hMnO*_x_* incubated MRSA biofilms with or without 808 nm laser (1.0 W cm^−2^) irradiation. Purple: ICG@hMnO*_x_*; green: MRSA biofilm. C) CLSM images, and D) the corresponding line profiles of fluorescence intensity across the white lines, suggesting the tight interaction between FITC‐stained MRSA and ICG@hMnO*_x_*. E) Membrane potential of MRSA upon indicated treatments was assessed by the DiOC_2_(3) staining. F) Fluorescence images of ROS marker DCFH‐DA stained MRSA upon indicated treatments. G) Schematic illustration of the highly efficient biofilm penetration, bacterial capture, and GSH depletion induced by ICG@hMnO*_x_* for photothermal‐boosted NCT against MRSA biofilm.

Next, the underlying mechanisms associated with the highly efficient anti‐bacterial and anti‐biofilm performance of ICG@hMnO*_x_* were explored. Manganese oxide nanoparticles (MONPs) with a smooth surface are synthesized according to our previous work to evaluate the morphology effect on biofilm penetration and bacterial capture capacities.^[^ ^24^ ^]^ As shown in Figure S14, Supporting Information, the smooth MONPs exhibit an enhanced biofilm penetration capacity, but their bacterial capture capacity is limited. In contrast to the smooth MONPs, the virus‐spiky ICG@hMnO*_x_* possesses a limited biofilm penetration but an enhanced bacterial capture capacity in the absence of laser irradiation. These findings suggest that there is a trade‐off limitation between the biofilm penetration and bacterial capture capacities of manganese nanoparticles with different surface morphologies. Excitingly, as evidenced by the crystal violet staining results, ICG@hMnO*_x_* treated MRSA biofilm exhibits a remarkable dissociation and integrity destruction after laser irradiation (**Figure** **4**A). A ROS inhibitor N‐acetylcysteine (NAC) was utilized to confirm whether oxidative damage is involved in the biofilm disruption process.^[^ ^25^ ^]^ Notably, there is no significant difference between ICG@hMnO*_x_* plus laser treated biofilm with or without NAC, indicating that the biofilm disruption is mainly caused by the photothermal effect rather than the ROS‐induced degradation of biofilm (Figure 4A). Next, the effect of photothermal on the capability of ICG@hMnO*_x_* to penetrate biofilm was further investigated. As shown in Figure 4B, the ICG@hMnO*_x_* remains mostly on the surface of MRSA biofilm without laser irradiation, and penetrates deeply inside the MRSA biofilm under laser exposure. Notably, the NAC treatment barely affects the biofilm penetration of ICG@hMnO*_x_* (Figure 4B), demonstrating that the photothermal effect of ICG@hMnO*_x_* is responsible for the effective biofilm disruption and penetration.^[^ ^18^ ^]^ Compared to the long time (up to 24 h) it takes for ROS to degrade bacterial biofilms for penetration,^[^ ^26^ ^]^ the thermally disrupting effect of ICG@hMnO*_x_* achieves a quick (≈30 min) and highly efficient biofilm penetration.

Considering the short lifetimes and limited action radius of ROS, the bacterial capture capacity is another decisive factor for the bactericidal effect of a nanozyme.^[^ ^11^ ^]^ Therefore, the bacterial capture capacity of ICG@hMnO*_x_* was further studied. As shown in Figure 4C,D, the well co‐localized purple fluorescence signals of ICG@hMnO*_x_* and the green fluorescence signals of fluorescein isothiocyanate (FITC)‐modified MRSA indicates the robust bacterial capture capacity of ICG@hMnO*_x_*. In addition, the virus‐spiky surface mediated bacterial capture was also investigated by TEM. As shown in Figure S15, Supporting Information, negligible bacterial capture was observed in the smooth SSN group. By comparison, the virus‐spiky ICG@hMnO*_x_* with a similar size to SSN can bind to the MRSA membrane efficiently, indicating that the virus‐spiky surface is beneficial for enhanced bacterial capture (Figure S15, Supporting Information). To evaluate the impact of virus‐spiky ICG@hMnO*_x_* on bacterial membrane, the membrane potential of MRSA was further detected by the fluorescent dye 3,3′‐diethyloxacarbocyanine iodide (DiOC_2_(3)).^[^ ^27^ ^]^ As shown in Figure 4E, the ICG@hMnO*_x_* treated MRSA exhibit a significantly decreased red/green fluorescence ratio, indicating a severe disruption of the bacterial membrane, a loss of membrane potential, and membrane depolarization mediated by ICG@hMnO*_x_*, verifying the essential role of the virus‐spiky surface in the capture and disruption of MRSA membrane. Consistently, the intracellular level of ROS is significantly elevated in the ICG@hMnO*_x_* treated bacteria coupled with irradiation, suggesting that ICG@hMnO*_x_* can serve as a membrane‐anchored ROS generator through the photothermal‐boosted POD‐like activity (Figure 4F, Figure S16A, Supporting Information). As an endogenous antioxidant defense system, GSH can prevent oxidative stress from damaging the bacterial cell and thus reduce the therapeutic effect of NCT.^[^ ^28^ ^]^ Therefore, the intracellular GSH level under different treatments was further investigated. Compared with the control groups, the intracellular GSH level of MRSA can be significantly down‐regulated by ICG@hMnO*_x_* with laser irradiation (Figure S16B, Supporting Information), suggesting that the photothermal effect can accelerate the GSH depletion by ICG@hMnO*_x_*. Overall, these results confirm a synergistic multiscale NCT against MRSA infections by using ICG@hMnO*_x_* (Figure 4G), including biofilm penetration (extracellular level), bacterial capture (cellular level), and GSH depletion (intracellular level).

### 2.4 In Vivo Photothermal‐Boosted NCT of MRSA Infections by ICG@hMnO*_x_*

**Figure 5:**
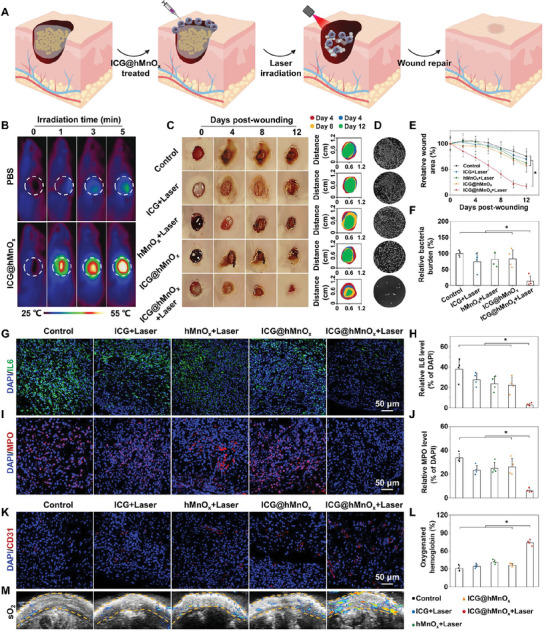
In vivo wound disinfection and healing by using the photomodulable bacteriophage‐spike ICG@hMnO*_x_*. A) Schematic illustration of the full‐thickness MRSA wound model and the in vivo antimicrobial activity of ICG@hMnO*_x_*. B) Real‐time thermal photographs of PBS‐ or ICG@hMnO*_x_*‐treated wound under irradiation with an 808 nm laser (1.5 W cm^−2^). C) Representative photographs and closing traces of the MRSA‐infected wound at 0, 4, 8, and 12 days post‐wounding after different treatments. D) MRSA colonies at 4 days post‐wounding with indicated treatments. E) Wound healing kinetics of different groups (*n* = 5). F) Relative bacterial burden of wound tissues harvested from different groups at 4 days post‐wounding (*n* = 4). G) Representative IL6 immunofluorescence staining images, and H) corresponding quantification of IL6 level in wound tissues harvested from different groups at 4 days post‐wounding (*n* = 4). I) Representative immunofluorescence images of MPO in wound tissues harvested from different groups at 4 days post‐wounding, where the red fluorescence indicates the expressed MPO in wound tissues. J) Quantitative analysis for immunofluorescence staining of MPO (*n* = 4). K) Immunofluorescence images of CD31 positive blood vasculature in wound tissues harvested from different groups at 14 days post‐wounding. L) Quantification of oxygen saturation (sO_2_) level in wound tissues at 14 days post‐wounding (*n* = 4). M) Photoacoustic maps of sO_2_ in wound tissues at 14 days post‐wounding. Data are presented as mean ± s.d. *p < 0.05.

A MRSA‐infected wound model was established to assess the in vivo therapeutic effects of ICG@hMnO*_x_* (**Figure** **5**A). As shown in Figure 5B, compared with the PBS treatment, a significant temperature elevation in wound tissue is achieved by ICG@hMnO*_x_* treatment with laser irradiation. To monitor the wound disinfection and healing capacity, photographs of the wounds under different treatments were recorded. Notably, the ICG@hMnO*_x_* plus laser irradiation treated wounds exhibit a much faster healing rate and less bacterial burden compared with the control groups (Figure 5C–F). These results demonstrate ICG@hMnO*_x_* coupled with laser irradiation can efficiently eliminate MRSA biofilm at the infected wound to promote wound healing. Moreover, the ICG@hMnO*_x_* plus laser irradiation significantly reduces the infiltration and invasion of MRSA to surrounding tissues (Figure S17, Supporting Information), demonstrating the superiority of ICG@hMnO*_x_* mediated photothermal‐boosted NCT in preventing MRSA infection.

Subsequently, wound repair and regeneration were investigated by assessing the inflammatory responses (including expression of myeloperoxidase (MPO) and pro‐inflammatory cytokine interleukin‐6 (IL‐6)) and vessel formation.^[^ ^29^ ^]^ The significantly reduced MPO and IL‐6 expression suggest the inflammatory responses in ICG@hMnO*_x_* plus laser irradiation treated wounds are significantly suppressed (Figure 5G–J). Remarkably, the immunofluorescence staining of angiogenic marker CD31 reveals a large amount of blood vessels in the ICG@hMnO*_x_* plus laser irradiation treated wounds (Figure 5K). Moreover, the significant improvement of blood oxygen saturation in the wound region indicates a promising effect of ICG@hMnO*_x_* to promote wound repair via facilitating the functional blood vessel mediated oxygen and nutrient supplementation (Figure 5L,M). Consistently, the ICG@hMnO*_x_* plus laser irradiation treated wounds show sufficient collagen deposition and skin appendages formation as evidenced by the Masson trichrome and hematoxylin‐eosin (H&E) staining (Figure S18, Supporting Information), and the expression of proliferative marker Ki67 is greatly enhanced in the wound tissue (Figure S19, Supporting Information). These results collectively suggest that the photothermal‐boosted NCT mediated by ICG@hMnO*_x_* can significantly promote wound repair and regeneration processes by eliminating MRSA biofilm infection, suppressing inflammatory responses, and facilitating angiogenesis. Additionally, the histopathological examination of main organs and metabolic assessment in mice demonstrates that ICG@hMnO*_x_* is highly biocompatible (Figure S20, Supporting Information). Besides, the Mn ion content in the newly healed wound tissue treated with ICG@hMnO*_x_* is similar to that of normal skin, indicating negligible accumulation of ICG@hMnO*_x_* (Figure S21, Supporting Information).

## 3 Conclusion

In this study, we presented a photomodulable bacteriophage‐spike nanozyme ICG@hMnO*_x_* by integrating ICG with a hollow virus‐spiky MnO*_x_* nanozyme. ICG@hMnO*_x_* exhibits superior photothermal performance for disrupting the compact biofilm and enhancing the efficiency of bactericidal POD‐mimetic reactions. Therefore, the ICG@hMnO*_x_* not only penetrates deep into biofilm by taking advantage of the ICG‐enabled photothermal effect, but also serves as a membrane anchored ROS‐generator benefitting from the bacterial adhesive virus‐spiky surface, resulting in the unprecedented combination of efficient biofilm penetration and bacterial capture. Notably, the ICG@hMnO*_x_* exhibits NIR‐augmented POD‐like catalytic activity and GSH depleting capacity, which greatly compromises the innate antioxidant defense of MRSA for photothermal‐boosted catalytic therapy. Consequently, ICG@hMnO*_x_* exerts a remarkable multiscale bactericidal NCT against MRSA biofilm‐associated infection, including biofilm penetration (extracellular level), bacterial capture (cellular level) and GSH depletion (intracellular level), which leads to improved therapeutic outcomes against MRSA infections both in vitro and in vivo. Furthermore, the NIR laser irradiation can precisely regulate the catalytic activity of ICG@hMnO*_x_*, offering a promising approach for the precise therapy of infectious diseases. The ICG@hMnO*_x_* achieves a precise nanocatalytic therapy against MRSA infections by combining dually‐enhanced biofilm penetration and bacterial capture with precisely tunable antibacterial activity and GSH depletion. These unique features distinguish ICG@hMnO*_x_* from state‐of‐the‐art antibacterial nanomaterials and provide new insights into the design of bactericidal nanozymes. Beyond the specific design proposed here, it is conceivable that this strategy could be extended to other nanozymes with different capture modes (such as electrostatic adsorption or bacteria‐targeting ligand modification) or other biofilm disruptors (such as magnetothermal or sonothermal disruption).

## 4 Experimental Section

### Materials

Tetraethoxysilane (TEOS), ammonium hydroxide (NH_3_·H_2_O), sodium carbonate (Na_2_CO_3_), indocyanine green (ICG), 3,3′,5,5′‐tetramethyl‐benzidine (TMB), hydrogen peroxide (H_2_O_2_, 30 wt%), fluorescein isothiocyanate (FITC), 2.5% glutaraldehyde, and N‐acetyl‐L‐cysteine (NAC) were obtained from Aladdin Reagent (Shanghai, China). Ethanol and potassium permanganate (KMnO_4_) were purchased from Sinopharm Chemical Reagent Co. Ltd (Beijing, China). 5,5′‐dithiobis‐2‐(nitrobenzoicacid) (DTNB) was purchased from Yeasen biotech Co. Ltd (Shanghai, China). Tryptone soy broth (TSB), Tryptone soy agar (TSA), 1% crystal violet dye, and phosphate‐buffered saline (PBS) were purchased from SolarBio Co. Ltd (Beijing, China). SYTO9 was brought from KeyGen Biotechnology Co. Ltd (Nanjing, China). 3,3′‐diethyloxacarbocyanine iodide (DiOC_2_(3)) was purchased from Macklin Biochemical Co. Ltd (Shanghai, China). The 5,5‐dimethyl‐1‐pyrroline N‐oxide (DMPO) was obtained from Dojindo Molecular Technologies, Inc (Dojindo, Japan). Propidium iodide (PI), ROS assay kit 2′‐7′‐dichlorofluorescin diacetate (DCFH‐DA), myeloperoxidase (MPO) antibody (AF7494), interleukin‐6 (IL‐6) antibody (AF7236), Ki67 antibody (AF1738), and platelet endothelial cell adhesion molecule‐1 (CD31) antibody (AF6408) were purchased from Beyotime Institute of Biotechnology (Haimen, China). All chemicals and reagents were used without further purification.

### Material Characterization

The morphology of nanoparticles and bacteria was imaged using an HT770 transmission electron microscopy (TEM, Hitachi, Japan). The surface microstructure of nanoparticles and bacteria were assessed by a Hitachi SU8010 field emission scanning electron microscopy (SEM, Hitachi, Japan). The high‐resolution transmission electron microscopy (HR‐TEM), selected area electron diffraction (SAED) pattern, and the scanning transmission electron microscopy‐energy dispersive X‐ray spectroscopy (STEM‐EDX) element distribution of ICG@hMnO*_x_* were characterized using a FEI Tecnai F20 (FEI, USA). The UV–visible absorption spectra of samples were obtained using a UV‐2600 spectrophotometer (Shimadzu, Japan). The N_2_ adsorption/desorption isotherms were collected on Autosorb‐iQ‐MP (Quantachrome Instruments, USA). The X‐ray powder diffraction (XRD) patterns were collected on a Rigaku D/Max‐2550 PC instrument (Rigaku, Japan). The X‐ray photoelectron spectra were recorded on an ESCALAB 250XI photoelectron spectrometer (ThermoFisher Scientific, USA). The EPR spectra were conducted with an EPR200M electron paramagnetic resonance spectrometer (CIQTEK, China). The size distributions were obtained using a Zetasizer Nano ZS90 (Malvern Instruments, UK). The absorbance of samples was measured using a Spectra Max 190 microplate reader (Molecular Devices, USA). The fluorescence images were collected by a FV1200 confocal laser scanning fluorescence microscope (CLSM, Olympus, Japan). Photoacoustic images were collected on a Vevo 2100 LAZR photoacoustic imaging system (Visualsonics, Canada).

### Fabrication of ICG@hMnO*_x_*

SSNs were first synthesized as the self‐sacrificing template by stöber method, using the TEOS as precursor.^[^ ^30^ ^]^ Briefly, 1 mL of NH_3_·H_2_O were first mixed with 40 mL of ethanol (95 wt%). After stirring for 30 min at 40 °C, 0.5 mL of TEOS was then added dropwise to the above mixture with magnetic stirring for 24 h to obtain SSNs. The resultant SSNs were purified by washing with deionized water and ethanol, and stored in deionized water for further use. Next, the hMnO*_x_* was synthesized according to a previous literature report.^[^ ^19^ ^]^ In a typical synthesis procedure, KMnO_4_ and SSNs were dissolved in deionized water, respectively. Next, 30 mL of KMnO_4_ solution (10 mg mL^−1^) was slowly added to 4 mL of SSN solution (10 mg mL^−1^) under ultrasonication. After 6 h of ultrasonication, the SSN@MnO*_x_* was obtained by centrifugation and washed for several times with ethanol and deionized water. To remove the core template SSN, the as‐prepared SSN@MnO*_x_* was dissolved in aqueous solution of Na_2_CO_3_ (2 m), and the resulting mixture was stirred for 12 h at 60 °C. Afterward, the brown‐black hMnO*_x_* was collected by centrifugation, washed several times with deionized water and ethanol to remove the remaining reactants. In a typical synthesis procedure, 10 mg of hMnO*_x_* NPs were added to PBS containing ICG (200 µg) and stirred overnight in darkness for ICG loading. The obtained ICG@hMnO*_x_* was then centrifuged to remove the remaining ICG and subsequently dried in a freezer dryer for further use, and the supernatant was collected for UV–vis analysis. The DLS size changes of the ICG@hMnO*_x_* in different physiological solutions, including DI water, PBS, and DMEM were recorded at 0, 24, 48, and 72 h. To evaluate the leakage of ICG during storage, fluorescence images of the initial ICG@hMnO*_x_* solution and corresponding centrifuged supernatant were recorded at 0 and 3 days, respectively. For comparison, MONP with smooth surface was also synthesized according to a previous literature report.^[^ ^24^ ^]^

### Photothermal activity of ICG@hMnO*_x_*

To investigate the photothermal capacity, aqueous dispersions of different nanoformulations with an equivalent ICG concentration (50 µg mL^−1^) were irradiated by an 808 nm NIR laser at a power density of 1.0 W cm^−2^ for 5 min. The corresponding temperature evolutions were recorded by thermocouple microprobe and infrared thermal imaging camera, respectively. Next, ICG@hMnO*_x_* with various concentrations (0, 12.5, 25, and 50 µg mL^−1^) of ICG was irradiated by an 808 nm NIR laser at a power density of 1.0 W cm^−2^ for 5 min. The solution temperature was monitored every 30 s by a thermocouple microprobe. The influence of laser power density on photothermal properties of ICG@hMnO*_x_* were next evaluated by exposing ICG@hMnO*_x_* to 808 nm NIR laser with different power densities (0, 0.5, 1.0, and 1.5 W cm^−2^) for 5 min. The solution temperatures were monitored every 30 s by a thermocouple microprobe. To study the photothermal stability of ICG@hMnO*_x_*, ICG@hMnO*_x_* and ICG, with an equivalent dose of 50 µg mL^−1^ ICG, were exposed to an 808 nm NIR laser irradiation at a power density of 1.0 W cm^−2^ for 8 min and then cooling to ambient temperature. The temperature of above solutions was measured by a thermocouple microprobe for three laser on‐off cycles.

### Photothermal‐Boosted Catalytic Activity of ICG@hMnO*_x_*

The POD‐like activity of ICG@hMnO*_x_* was evaluated by using the classic colorimetric analysis based on the oxidation of TMB. In brief, aqueous dispersions of different nanoformulations were added to 1 mL of PBS buffer (pH 6.5) containing TMB (0.5 mm) and H_2_O_2_ (1.0 mm) with or without laser irradiation (808 nm, 1.0 W cm^−2^). Next, to evaluate the effect of laser power density on the POD‐like activity, the ICG@hMnO*_x_* were added to 1 mL of PBS buffer (pH 6.5) containing TMB (0.5 mm) and H_2_O_2_ (1.0 mm), followed by exposure to 808 nm NIR laser with different power densities (0, 0.5, 1.0, and 1.5 W cm^−2^). Meanwhile, the POD‐like activity of ICG@hMnO*_x_* nanozymes at different temperatures (25, 30, 35, and 40 °C) were also performed. The absorbance spectra of the above solutions were recorded by a UV–vis spectrophotometer.

### Planktonic Bacteria and Biofilm Culture

Methicillin‐resistant *Staphylococcus aureus* (MRSA) was selected as the model bacterium to evaluate the antibacterial performance ICG@hMnO*_x_*, which was cultured in TSB medium or Tryptone soy agar (TSA) plates. Typically, single colony of MRSA was transplanted to TSB medium and grown at 37 °C with shaking (200 rpm) overnight for subsequent investigations on planktonic bacteria. For the biofilm formation, bacteria suspensions (1 × 10^8^ CFU mL^−1^) in TSB medium were cultured in sterile plates at 37 °C for 48 h, and the medium was changed once a day. Thereafter, the medium was removed and washed slightly with PBS to discard unattached MRSA.

### Antibacterial Activity of ICG@hMnO*_x_* In Vitro

To study the in vitro antibacterial effects, exponentially growing MRSA was first harvested and diluted to 1 × 10^6^ CFU mL^−1^. Then, six experimental groups were set up in the absence and presence of laser irradiation, including the blank control group, H_2_O_2_ group, ICG@hMnO*_x_* + H_2_O_2_ group, ICG + H_2_O_2_ + Laser group, hMnO*_x_* + H_2_O_2_ + Laser group, and ICG@hMnO*_x_* + H_2_O_2_ + Laser group. The laser irradiation parameter was set as 1.0 W cm^−2^ for 2 min. After 12 h treatments, the diluted bacterial suspensions were harvested and spread on TSA plates and incubated overnight at 37 °C. Thereafter, the colony of bacteria was analyzed and photographed. For live/dead fluorescent staining, the treated bacteria were harvested by centrifugation and washed with PBS. After that, the bacteria were incubated with SYTO9 and PI solution in darkness for 30 min according to the instructions of staining kit. The bacteria were then visualized with the laser scanning confocal microscopy. To evaluate the susceptibility of MRSA to ICG@hMnO*_x_*, minimum inhibitory concentrations (MIC) and minimal bactericidal concentrations (MBC) were determined. To determine the MIC, serial twofold dilutions of ICG@hMnO*_x_* ranging from 3.13 to 200 µg mL^−1^ was incubated with MRSA bacteria for 12 h at 200 rpm. Subsequently, the treated MRSA were transferred to sterile agar plates to determine the MBC. The laser irradiation parameter was set as 1.0 W cm^−2^, 5 min for MIC and MBC evaluation. To evaluate the bacterial morphology changes, the treated bacteria suspensions were fixed with 2.5% glutaraldehyde solution for 12 h at 4 °C. Thereafter, the fixed bacteria were sequentially dehydrated in a gradient of ethanol/water solution, followed by scanning electron microscope observation.

### In Vitro Colocalization Assay

Exponentially growing bacteria were harvested and incubated with the PBS or different nanoformulations (SSN or ICG@hMnO*_x_*) for 1 h and then fixed with 2.5% glutaraldehyde at 4 °C overnight. Next, the samples were examined by TEM analysis. In the fluorescent labeling experiment, MRSA was first tagged with FITC (5 µg mL^−1^, 2 h) and then washed with PBS for three times to remove the excess free FITC. Next, the FITC‐tagged bacteria were incubated with ICG@hMnO*_x_*. Then, the bacteria were washed with PBS buffer and fixed with 2.5% glutaraldehyde for CLSM observation.

### Membrane Disruption Activity of ICG@hMnO*_x_*

Fluorescent carbocyanine dye DiOC_2_(3) was used to assess the membrane potential of bacteria, in which the bacteria with intact cell membrane potential emit red fluorescence, whereas the loss of membrane potential leads to the emission of green fluorescence. The treated bacteria were harvested by centrifugation (4000 rpm, 3 min) and stained with 30 µm of DiOC_2_(3) for 30 min in the dark. Thereafter, the stained bacteria were observed by a confocal laser scanning microscope.

### Intracellular Reactive Oxygen Species Level

The intracellular ROS content in treated bacteria was monitored by the ROS‐sensitive DCFH‐DA fluorescent probe. After different treatments mentioned above, the treated bacteria were collected and incubated with 10 µM of DCFH‐DA probe at 37 °C for 20 min in the dark. Thereafter, the fluorescence intensity of stained bacteria was evaluated by confocal laser scanning microscope and flow cytometry. Next, the intracellular glutathione (GSH) content in the treated bacteria was evaluated by the DTNB. After treatments with different groups as mentioned above, 500 µL of the lysate of treated bacteria was collected and incubated with 5 µL of the DTNB (10 mg mL^−1^) for 60 min at 37 °C. Then, the amount of GSH was measured by recording the absorbance at 412 nm on a microplate reader.

### Antibiofilm Activity of ICG@hMnO*_x_* In Vitro

The biofilms were cultured as described above and subjected to various treatments (control, H_2_O_2_, ICG@hMnO*_x_* + H_2_O_2_, ICG + H_2_O_2_ + Laser, hMnO*_x_* + H_2_O_2_ + Laser, and ICG@hMnO*_x_* + H_2_O_2_ + Laser). The irradiation parameter in all the laser group was set as 1.0 W cm^−2^ for 2 min. Next, the live/dead staining kit was used to evaluate the treated biofilm. Briefly, as‐treated biofilms were incubated with the mixture of STYO9 and PI solution in darkness for 30 min. Subsequently, the stained biofilms were gently rinsed with PBS buffer to remove excess dyes and observed with CLSM. For quantitative analysis of the antibiofilm effect, the treated biofilms were stained with 1% crystal violet dye for 15 min. Next, the biofilms were carefully rinsed with PBS buffer and 150 µL of ethanol was added to each well to extract the crystal violet. To evaluate the biofilm biomass, the absorbance of extractions was recorded by a microplate reader at 595 nm.

### Biofilm Disruption and Penetration Analysis

To study the biofilm penetration performance of ICG@hMnO*_x_*, the cultured MRSA biofilms were tagged with the green fluorescence probe SYTO9. ICG was used as the red fluorescent probe to investigate the biofilm penetration capacity of ICG@hMnO*_x_*. Briefly, ICG@hMnO*_x_* solution was incubated with the MRSA biofilms in the absence and presence of laser irradiation (1.0 W cm^−2^, 2 min). Next, the treated biofilms were gently rinsed with PBS buffer and incubated with SYTO9 (20 µm) solution for 30 min in the dark. Additionally, to disclose the main mechanism of biofilm penetration, the biofilms with ICG@hMnO*_x_* + Laser treatment were pre‐incubated with the ROS scavenger NAC (20 mm). Subsequently, biofilm penetration depth from each group was observed with CLSM. Meanwhile, MRSA biofilms were treated with the same method described above and divided into three groups: ICG@hMnO*_x_*, ICG@hMnO*_x_* + laser, and ICG@hMnO*_x_* + laser + NAC group. To evaluate the structural integrity, treated biofilm from each group was stained with 1% crystal violet dye for 15 min, and then observed by a digital microscope.

### In Vitro Biocompatibility Assessment

Human immortalized HaCaT keratinocytes were used for in vitro biocompatibility experiments. The HaCaT cells were cultured by the DMEM medium containing 10% fetal bovine serum (FBS) and 1% penicillin/streptomycin in an incubator at 37 °C with 5% CO_2_. After incubated with ICG@hMnO*_x_* (0, 10, 20, 30, 40, and 50 µg mL^−1^) for 1 or 3 d, viability of HaCaT cells was evaluated using a CellTiter 96 AQueous One Solution Assay kit (MTS, Promega).

The hemolytic activity of ICG@hMnO*_x_* was evaluated by using red blood cells (RBCs) from fresh mice blood. The diluted RBCs were incubated with Triton X‐100, saline solution and ICG@hMnO*_x_* (50 µg mL^−1^) at 37 °C for 1 h and photographed subsequently.

### In Vivo Antibacterial Studies

All the animal experiments for this study were approved by the Animal Care and Experiment Committee of the Zhejiang University (approval number: SYXK (Zhejiang) 2018‐0016). BALB/c mice (6–8 weeks old) were purchased from Shanghai Slac Laboratory Animal Co., Ltd. and raised in the specific‐pathogen‐free (SPF) grade laboratory. Briefly, mice were anesthetized with isoflurane and an 8 mm‐diameter full thickness circular wound seeded with MRSA (1 × 10^8^ CFU mL^−1^, 20 µL) was made on the back of each mouse by a sterile surgical scissor. Next, the infected mice were randomly divided into 5 groups and treated with 50 µL of different dispersions in blank control, ICG + Laser, hMnO*_x_* + Laser, ICG@hMnO*_x_*, and ICG@hMnO*_x_* + Laser, respectively. The irradiation parameter in all the laser group was set as 1.5 W cm^−2^ for 5 min. During laser irradiation, the time‐dependent temperature changes was monitored by an infrared camera. Additionally, the changes of wound area were photographed and quantified by the ImageJ software (National Institutes of Health, Bethesda, MD) every other day during the healing process. On day 4 post‐wounding, the infected skins were harvested and homogenized. Subsequently, the diluted homogenates were and spread on TSA plates and incubated overnight at 37 °C. Then the TSA plates were photographed and the colony of bacteria was counted. Meanwhile, the day 4 wounded tissues were fixed in 4% formalin solution and subjected to Gram's staining for evaluation bacterial burden. Moreover, to evaluate the inflammatory responses in day 4 wounded tissues, MPO and IL‐6 in the wound tissues were analyzed by immunofluorescence staining. On day 14 post‐wounding, the treated wounds were collected and fixed with 4% formalin solution for histological H&E, Masson's trichrome, Ki67, and CD31 staining analysis. The paraffin sections of wound tissue were used for Gram's staining, H&E staining, Masson's trichrome staining, and immunofluorescence staining by the Pathology Laboratory at Zhejiang University. The Gram, H&E, and Masson's trichrome staining images were obtained by optical microscope. The MPO, IL6, Ki67, and CD31 immunofluorescence images were obtained by a confocal laser scanning microscope. Additionally, the blood oxygen saturation (sO_2_) level on wounded tissues at day 14 post‐wounding was evaluated by a Vevo 2100 LAZR photoacoustic imaging system. To evaluate the in vivo biosafety, major organs including heart, liver, spleen, lung, and kidney harvested at day 14 post‐wounding were subjected to H&E staining. The plasma level of aspartate transaminase (AST), alanine transaminase (ALT), blood urea nitrogen (BUN), creatinine (CRE), and uric acid (UA) from mice were also evaluated. The tissue accumulation of ICG@hMnO*_x_* was quantified by the inductively coupled plasma mass spectroscopy (ICP‐MS).

### Statistical Analysis

The results were presented as mean ± s.d. (*n* ≥ 3). Data analysis was conducted by using the Image J (version 1.8.0) and Origin 2019. The data normalization, number of samples in each group (*n*), probability (*p*) value, and statistical test for each data were demonstrated in the figure legends. The significant differences between groups were analyzed through one‐way or two‐way analysis of variance (ANOVA) with Tukey's post hoc test and a *p* value <0.05 considered to be statistically significant.

## Conflict of Interest

The authors declare no conflict of interest.

## Supporting information

Supporting Information

## Data Availability

The data that support the findings of this study are available on request from the corresponding author. The data are not publicly available due to privacy or ethical restrictions.
