# Supplementary material for: A Photomodulable Bacteriophage‐Spike Nanozyme Enables Dually Enhanced Biofilm Penetration and Bacterial Capture for Photothermal‐Boosted Catalytic Therapy of MRSA Infections
Source: Adv Sci (Weinh). 2023 Jun 13;10(24):2301694. doi: 10.1002/advs.202301694 (PMC10460864; doi:10.1002/advs.202301694)
Supplement: Supplementary file 1 — Supporting Information [file ADVS-10-2301694-s001.pdf]

ADVANCED SCIENCE
Open Access

Supporting Information

for *Adv. Sci.*, DOI 10.1002/advs.202301694

A Photomodulable Bacteriophage-Spike Nanozyme Enables Dually Enhanced Biofilm Penetration and Bacterial Capture for Photothermal-Boosted Catalytic Therapy of MRSA Infections

Haibin Wu, Min Wei, Shen Hu, Pu Cheng, Shuhan Shi, Fan Xia, Lenan Xu, Lina Yin, Guang Liang, Fangyuan Li* and Daishun Ling**

Supporting Information

A Photomodulable Bacteriophage-Spike Nanozyme Enables Dually Enhanced Biofilm Penetration and Bacterial Capture for Photothermal-Boosted Catalytic Therapy of MRSA Infections

Haibin Wu, Min Wei, Shen Hu, Pu Cheng, Shuhan Shi, Fan Xia, Lenan Xu, Lina Yin, Guang Liang, Fangyuan Li*, Daishun Ling**

H. Wu, S. Shi, L. Xu, L. Yin, Prof. G. Liang
School of Pharmaceutical Sciences, Hangzhou Medical College, Hangzhou 311399, P. R. China
E-mail: wzmcliangguang@163.com (G. Liang)

M. Wei, F. Xia, Prof. F. Li
Institute of Pharmaceutics, College of Pharmaceutical Sciences, Zhejiang University, Hangzhou 310058, P. R. China
E-mail: lfy@zju.edu.cn (F. Li)

S. Hu, P. Cheng
Department of Obstetrics, The Second Affiliated Hospital, School of Medicine, Zhejiang University, Hangzhou 310009, P. R. China

Prof. D. Ling
Frontiers Science Center for Transformative Molecules, School of Chemistry and Chemical Engineering, National Center for Translational Medicine, Shanghai Jiao Tong University, Shanghai 200240, P. R. China
E-mail: dsling@sjtu.edu.cn (D. Ling), ORCID: 0000-0002-9977-0237 (D. Ling)

Prof. F. Li, Prof. D. Ling
Institute of Innovative Medicine, College of Pharmaceutical Sciences, Zhejiang University, Hangzhou 310012, P. R. China

Prof. F. Li, Prof. D. Ling
World Laureates Association (WLA) Laboratories, Shanghai 201203, P. R. China

Supplementary Figures

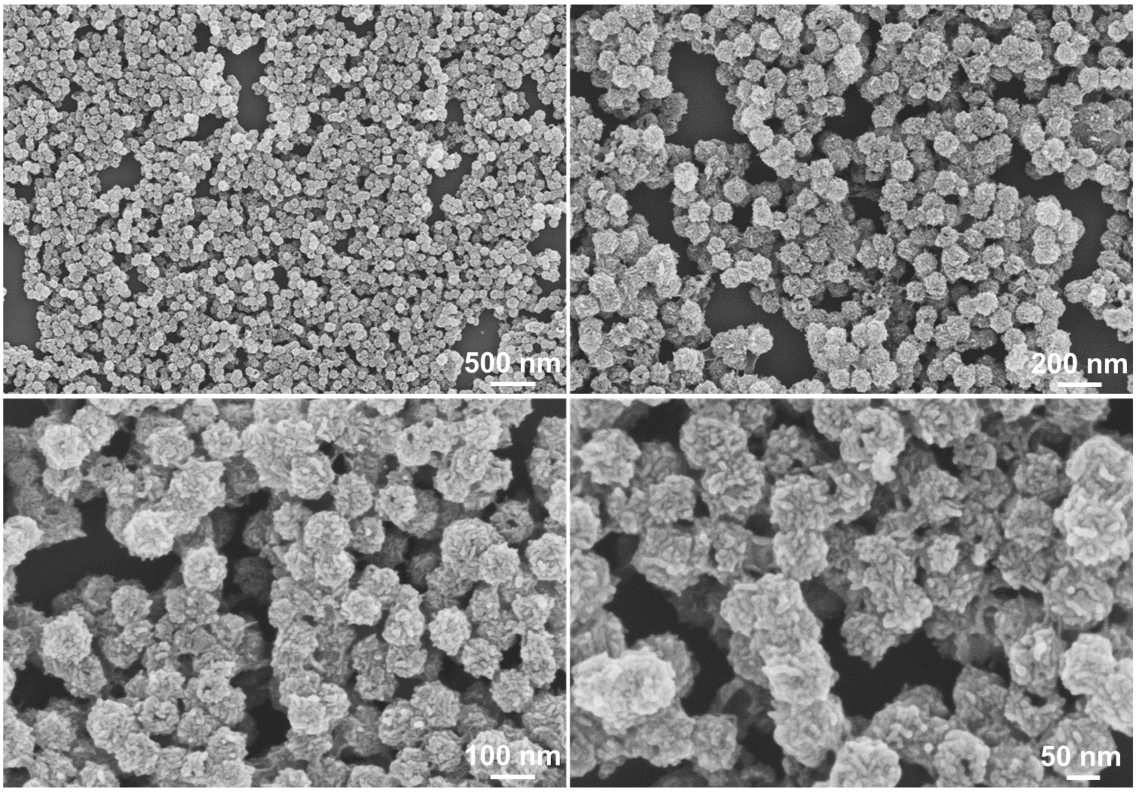

Figure S1. Representative scanning electron microscopy (SEM) images of ICG@hMnO$_x$.

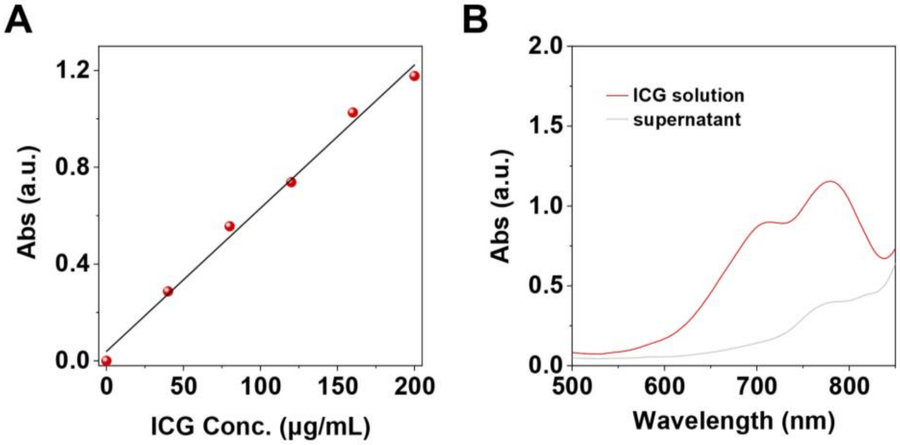

Figure S2. A) Standard curve for ICG solution obtained at the wavelength of 800 nm. **B**) The UV-vis spectra of the initial ICG solution and the centrifuged supernatant after the loading into hMnO$_x$.

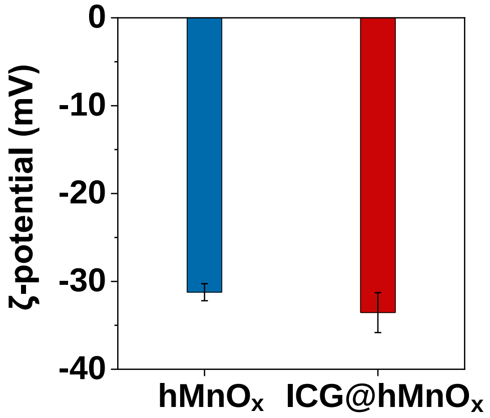

Figure S3. ζ-potentials of ICG@$hMnO_x$ and $hMnO_x$ (n = 4). Data are presented as mean ± s.d.

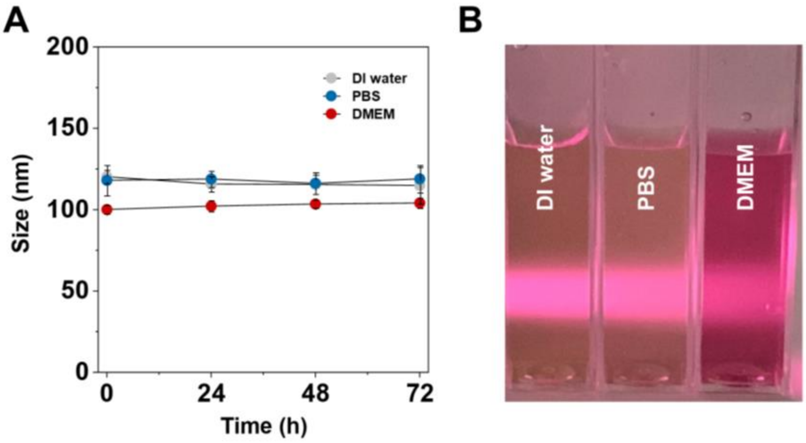

Figure S4. A) DLS profile of ICG@$hMnO_x$ in different physiological solutions (n = 4). **B**) Tyndall effect of ICG@$hMnO_x$ in different physiological solutions. Data are presented as mean ± s.d.

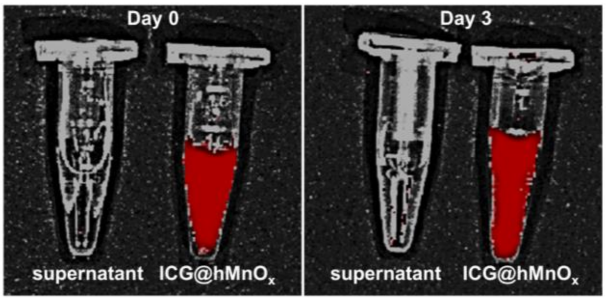

Figure S5. Fluorescence images of the initial ICG@hMnO_x solution and corresponding centrifuged supernatants before and after the 3-day storage.

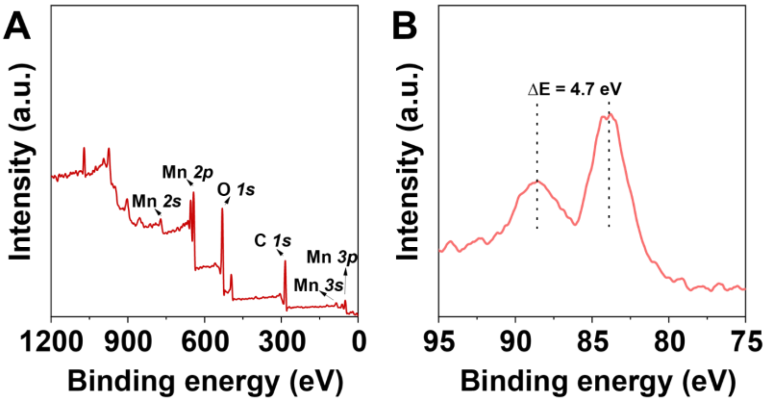

Figure S6. A) The X-ray photoelectron spectroscopy (XPS) spectrum of the ICG@hMnO_x. **B**) The high-resolution XPS spectrum of *Mn 3s* in ICG@hMnO_x.

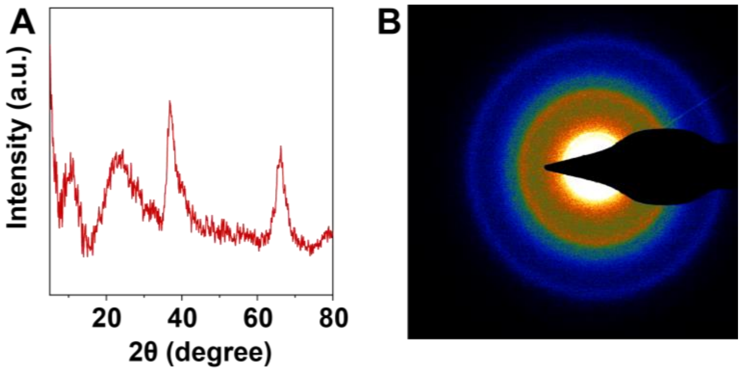

Figure S7. **A**) The XRD spectrum of the ICG@hMnO$_x$. **B**) SAED pattern of the ICG@hMnO$_x$.

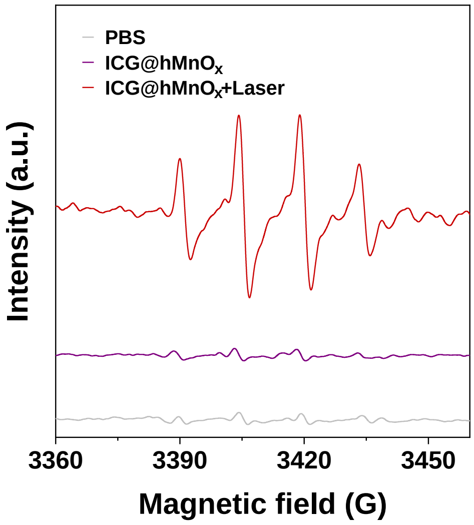

Figure S8. EPR spectra of DMPO-•OH under indicated treatments.

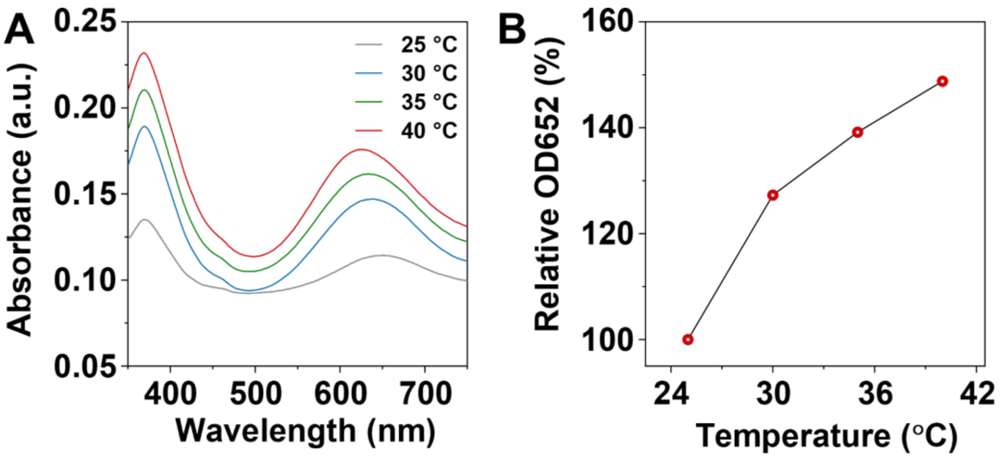

Figure S9. A) Ultraviolet and visible (UV-vis) spectra of 3,3′,5,5′-tetramethylbenzidine (TMB) solution incubated with ICG@hMnO$_x$ under different reaction temperatures. **B**) Temperature-dependent evolution of TMB absorbance at 652 nm.

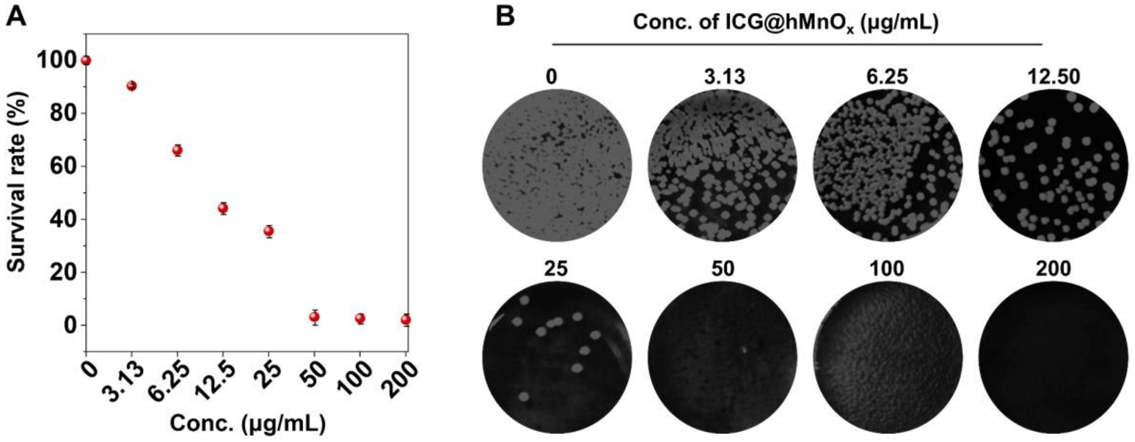

Figure S10. A) The survival rate of MRSA exposed to different concentrations of ICG@hMnO$_x$ with 808 nm laser (1.0 W/cm^2, 5 min) irradiation (n = 4). Data are presented as mean ± s.d. **B**) Representative plates of MRSA exposed to different concentrations of ICG@hMnO$_x$ with 808 nm laser (1.0 W/cm^2, 5 min) irradiation.

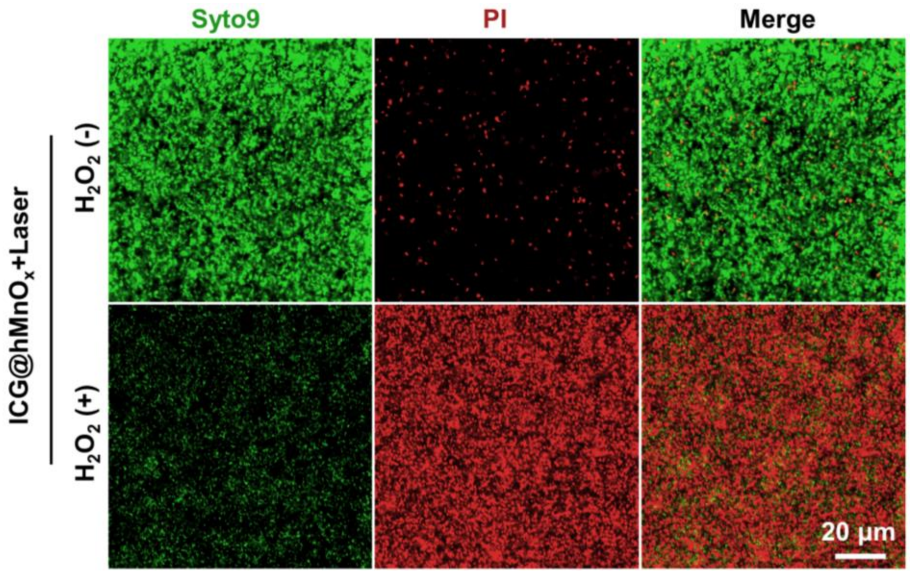

Figure S11. Live/dead staining of MRSA with or without POD reaction substrate H_2O_2.

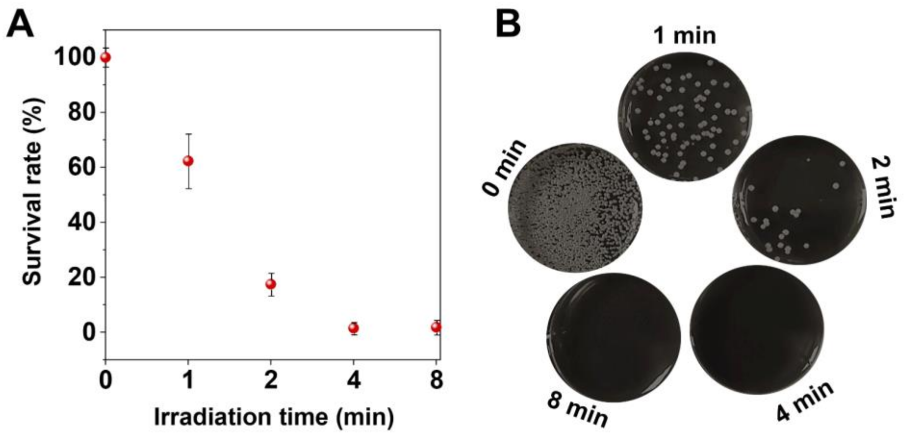

Figure S12. **A**) The survival rate of MRSA incubated with ICG@hMnO$_x$ for indicated irradiation time by 808 nm laser (n = 4). Data are presented as mean ± s.d. **B**) Representative plates of MRSA incubated with ICG@hMnO$_x$ for indicated irradiation time by 808 nm laser.

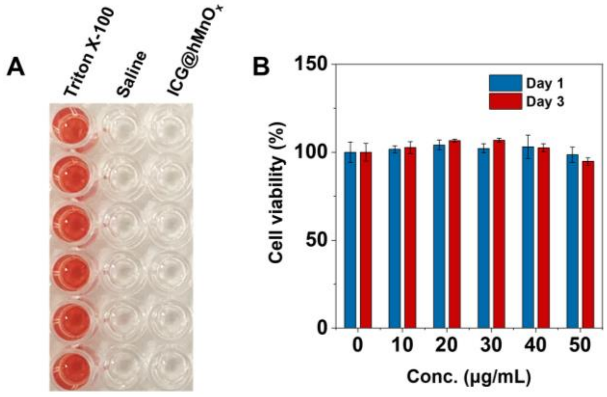

Figure S13. A) Representative photograph of hemolytic assay of indicated samples. **B**) Viability of HaCaT cells treated with indicated concentrations of ICG@hMnO_x (n = 4). Data are presented as mean ± s.d.

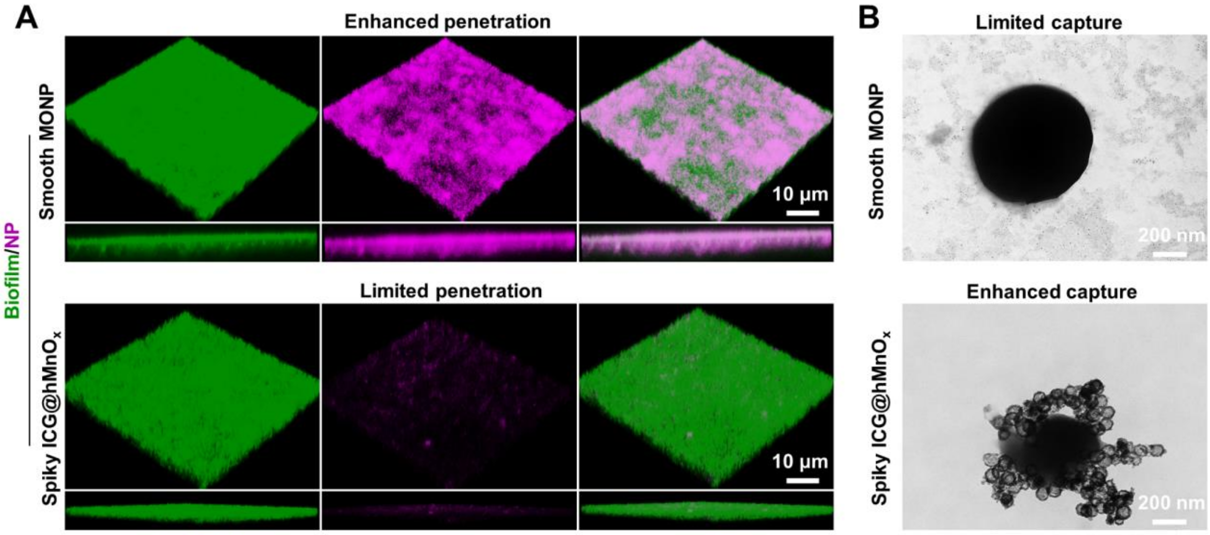

Figure S14. Trade-off between the biofilm penetration and bacterial capture capacity of manganese nanoparticles with different surface morphology. **A**) 3D CLSM images of the spiky ICG@hMnO_x or smooth ICG-MONPs incubated MRSA biofilms. Purple: ICG from nano-formulation; green: MRSA biofilm. **B**) TEM images of planktonic methicillin-resistant *Staphylococcus aureus* (MRSA) bacteria incubated with the spiky ICG@hMnO_x or smooth MONPs.

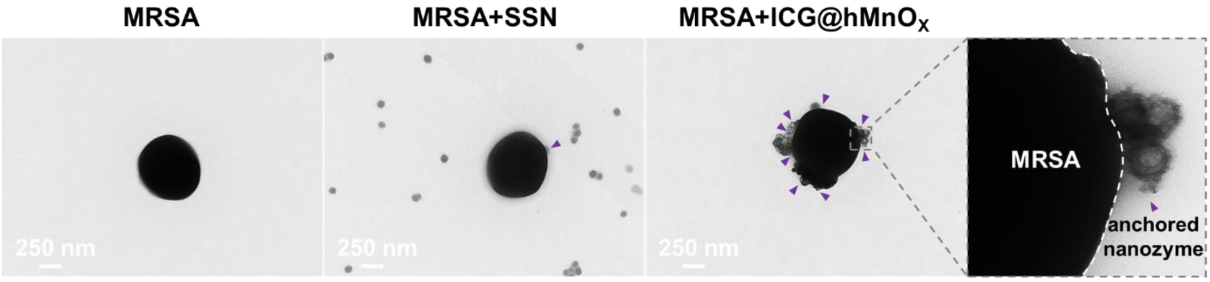

Figure S15. Representative TEM images of methicillin-resistant *Staphylococcus aureus* (MRSA) incubated with the smooth stöber silica nanoparticles (SSNs) and spiky ICG@hMnO$_x$, respectively.

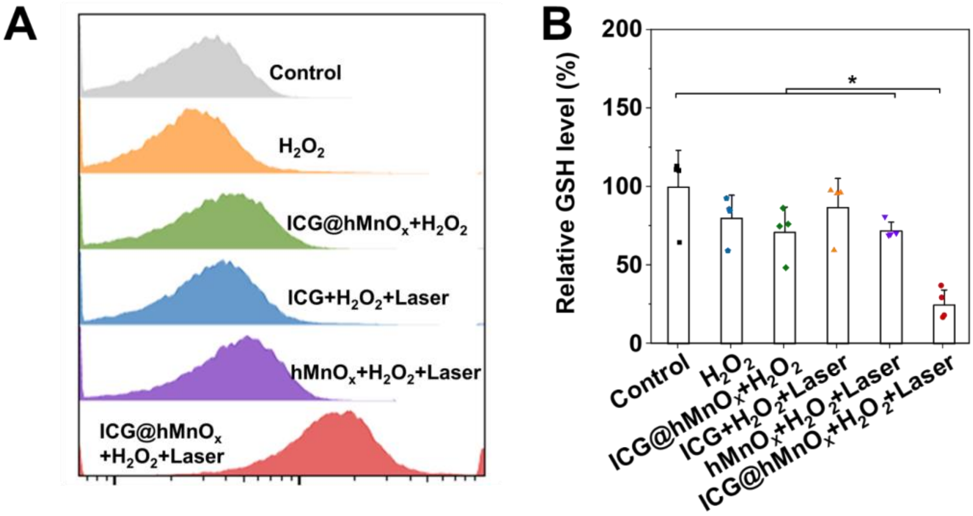

Figure S16. **A**) Flow cytometry analysis of reactive oxygen species (ROS) level in MRSA after different treatments. **B**) Relative glutathione (GSH) levels of MRSA after different treatments (n = 4). Data are presented as mean ± s.d. *$P < 0.05$.

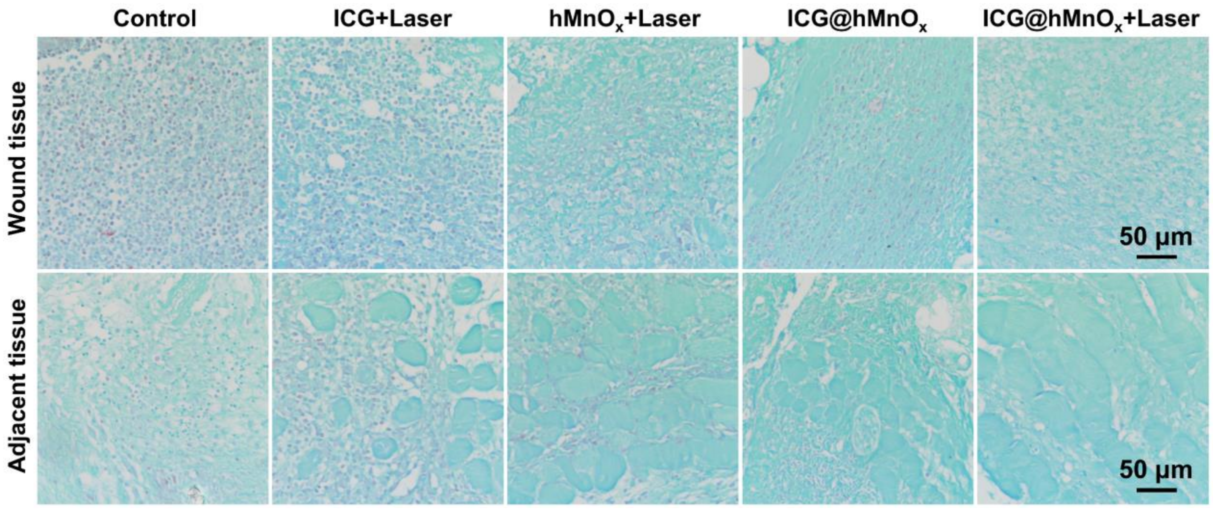

Figure S17. Images of Gram's stained wound tissues harvested from different groups at 4 d post-wounding.

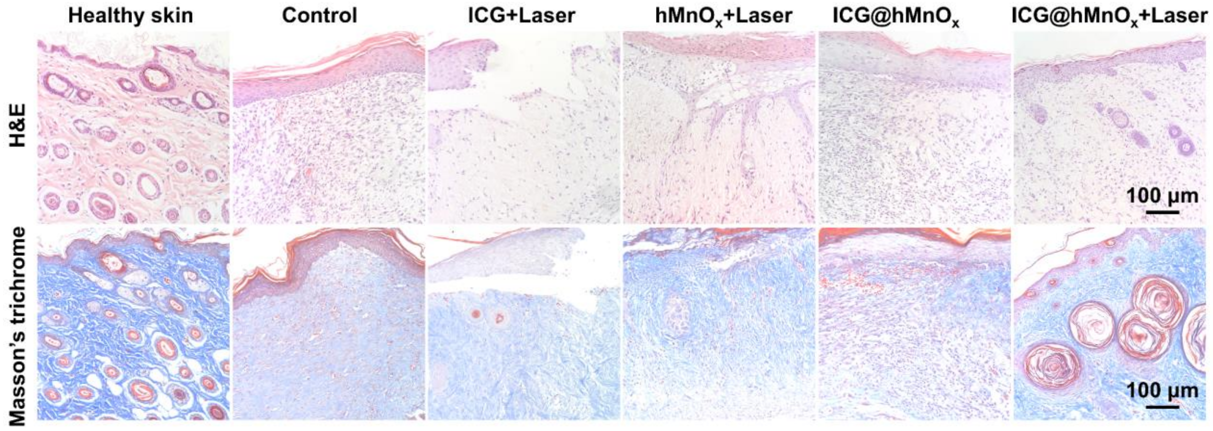

Figure S18. Images of hematoxylin-eosin (H&E) and Masson's trichrome stained wound tissues harvested from different groups at 14 d post-wounding.

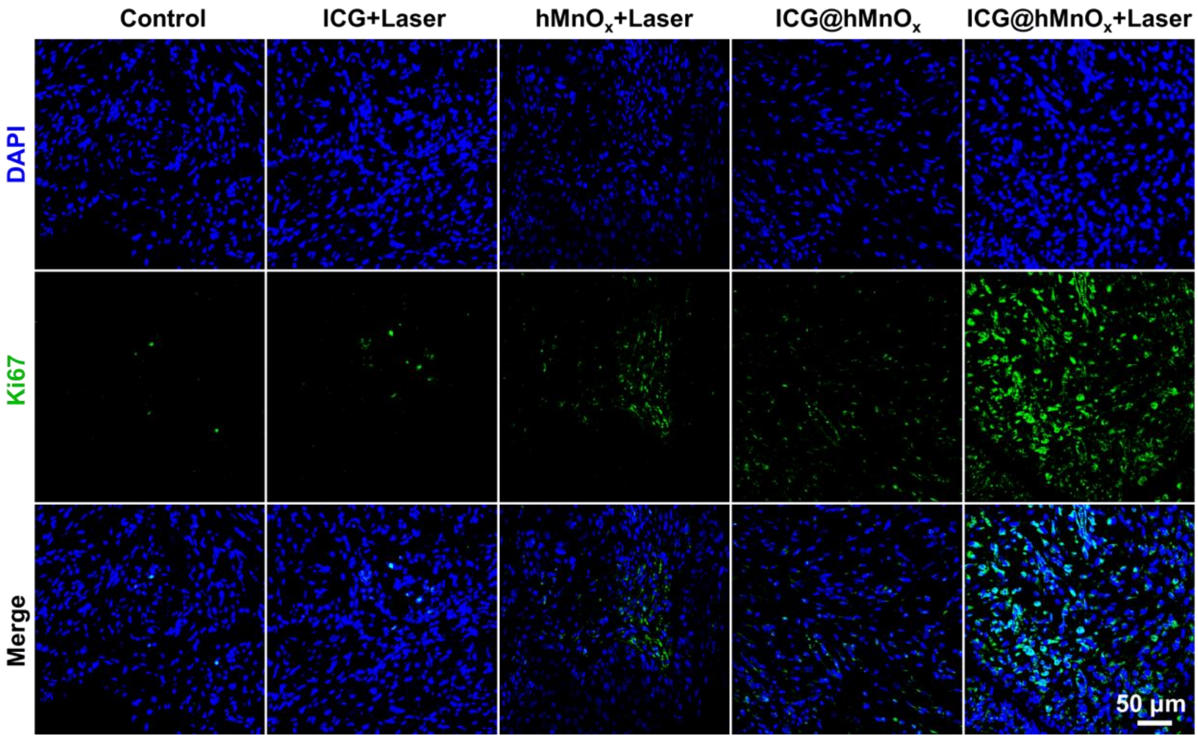

Figure S19. Representative immunofluorescence images of Ki67 in wound tissues harvested from different groups at 14 d post-wounding; green fluorescence indicates the expressed Ki67 in wound tissues.

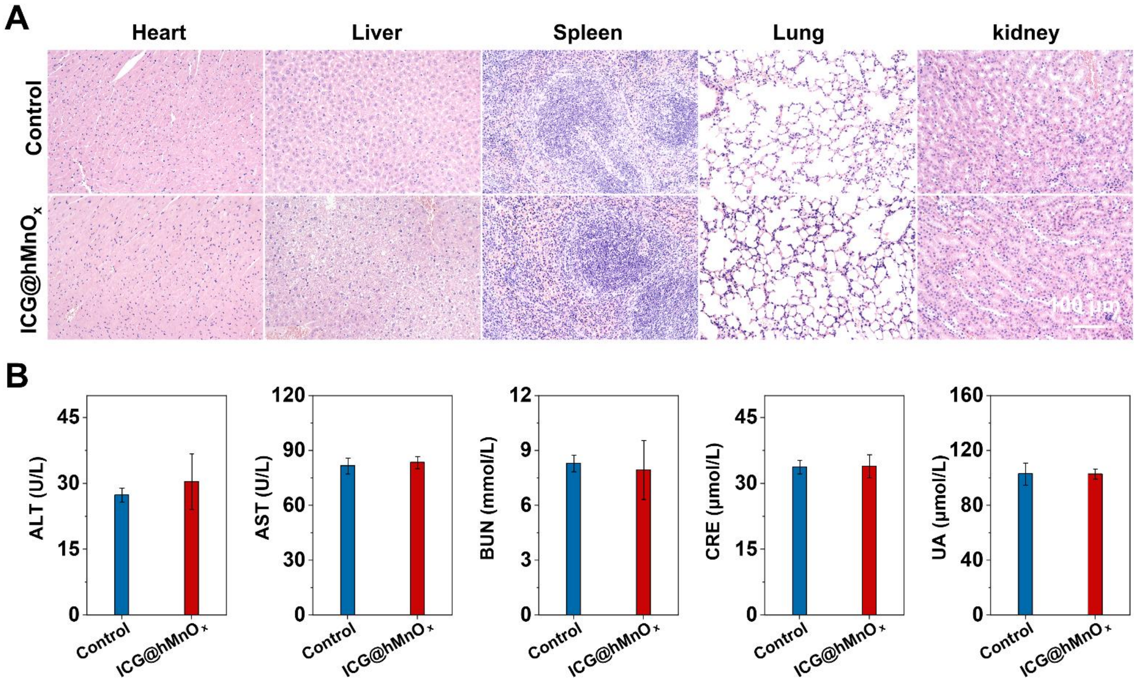

Figure S20. **A**) Representative H&E staining of major organs from mice treated with blank control or ICG@hMnO$_x$. **B**) Plasma level of aspartate transaminase (AST), alanine transaminase (ALT), blood urea nitrogen (BUN), creatinine (CRE), and uric acid (UA) from mice treated with blank control or ICG@hMnO$_x$ (n = 3). Data are presented as mean ± s.d.

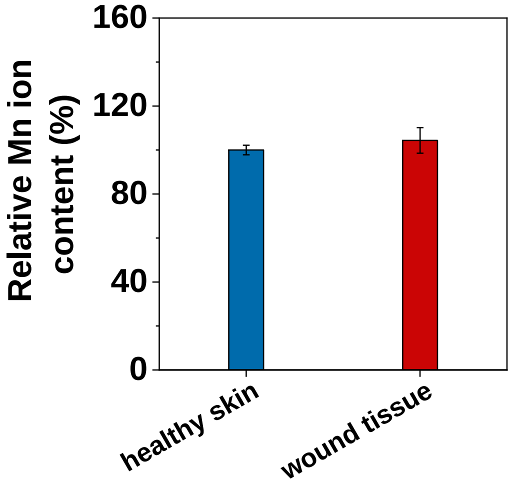

Figure S21. The Mn ion content in the newly healed wound tissue and healthy skin (n = 3). Data are presented as mean ± s.d.
